# Exploring the Interplay Between Language Comprehension and Cortical Tracking: The Bilingual Test Case

**DOI:** 10.1162/nol_a_00141

**Published:** 2024-06-03

**Authors:** Cristina Baus, Iris Millan, Xuanyi Jessica Chen, Esti Blanco-Elorrieta

**Affiliations:** Department of Cognition, Development and Educational Psychology, University of Barcelona, Barcelona, Spain; Institute of Neurosciences, University of Barcelona, Barcelona, Spain; Department of Psychology, New York University, New York, NY, USA; Department of Neural Science, New York University, New York, NY, USA

**Keywords:** bilingualism, entrainment, cortical tracking, language comprehension, N400, neural oscillations

## Abstract

Cortical tracking, the synchronization of brain activity to linguistic rhythms is a well-established phenomenon. However, its nature has been heavily contested: Is it purely epiphenomenal or does it play a fundamental role in speech comprehension? Previous research has used intelligibility manipulations to examine this topic. Here, we instead varied listeners’ language comprehension skills while keeping the auditory stimulus constant. To do so, we tested 22 native English speakers and 22 Spanish/Catalan bilinguals learning English as a second language (SL) in an EEG cortical entrainment experiment and correlated the responses with the magnitude of the N400 component of a semantic comprehension task. As expected, native listeners effectively tracked sentential, phrasal, and syllabic linguistic structures. In contrast, SL listeners exhibited limitations in tracking sentential structures but successfully tracked phrasal and syllabic rhythms. Importantly, the amplitude of the neural entrainment correlated with the amplitude of the detection of semantic incongruities in SLs, showing a direct connection between tracking and the ability to understand speech. Together, these findings shed light on the interplay between language comprehension and cortical tracking, to identify neural entrainment as a fundamental principle for speech comprehension.

## INTRODUCTION

Understanding how humans derive meaning from a continuous stream of sounds is one of the fundamental questions of human cognition. For this process to unfold successfully, listeners minimally need to segment the continuous speech stream into phonemes, subsequently reassemble such phonemes into words, and combine the resulting linguistic structures to build up a representation of the whole meaning. Hence, these operations involve lower-order (e.g., acoustic and phonetic) and higher-order (e.g., syntactic and semantic) representations, which are then coordinated to result in successful language comprehension.

Cortical entrainment to the temporal envelope of speech has recently been proposed to play an important role in this process, suggesting that neural tracking of speech features plays a pivotal role for the segmentation of syllabic and phrasal levels (Greenberg et al., 2003), and decoding and transforming the rhythms of speech into linguistic structures (Poeppel & Assaneo, 2020) through a combination of bottom-up and top-down processes (Giraud & Poeppel, 2012; Peelle & Davis, 2012; see Poeppel & Assaneo, 2020, for a review). However, there has been an ongoing debate about the precise mechanistic role of neural tracking in speech processing (Ding & Simon, 2014; Doelling & Assaneo, 2021; Kösem & van Wassenhove, 2017; Lakatos et al., 2019; Obleser & Kayser, 2019).

For instance, delta oscillations (1–4 Hz) have been proposed to reflect encoding of abstract syntactic structures; theta oscillations (4–8 Hz) are associated with syllables, words, and speech rhythmicity; and beta (15–30 Hz) and gamma (>50 Hz) bands are linked with phonological, lexical, and syntactic information (Ghitza, 2011; Kösem & van Wassenhove, 2017). Further, it is hypothesized that neural tracking at the mentioned frequencies might allow for better comprehension of the speech input (Blanco-Elorrieta et al., 2020; Zoefel et al., 2018).

Typically, to assess the relation between neural oscillations and language comprehension, experimental designs have compared the neural response to speech with that of an unintelligible *control* signal. This control signal is often generated by modifying the acoustic characteristics of clear speech, such as temporally reversing the speech signal or altering its temporal properties (Ahissar et al., 2001; Di Liberto et al., 2015; Doelling et al., 2014; Gross et al., 2013; Hincapié-Casas et al., 2021). Modifications may also include degrading spectral resolution (Chen et al., 2023; Hincapié-Casas et al., 2021; Meng et al., 2021; Molinaro & Lizarazu, 2018; Peelle et al., 2013) or altering the auditory background (Ding & Simon, 2013; Rimmele et al., 2015; Zion Golumbic et al., 2013; Zoefel & VanRullen, 2015a).

An alternative approach to establishing the relation between oscillations and language comprehension is to keep the speech signal clear but vary the level of linguistic understanding of the listener. Following this approach, previous research has shown that language knowledge is necessary for the brain to be able to segment speech into meaningful units (Ding et al., 2016; Ding et al., 2017). In their study, synthesized monosyllabic words were presented at 4 Hz. Importantly, each pair of two words constituted a phrase (presented at 2 Hz), and each pair of two-word phrases constituted a sentence (presented at 1 Hz). The study was conducted with native Mandarin Chinese speakers and native speakers of American English with no knowledge of Chinese. The results showed that in addition to entraining to the syllabic rhythm (4 Hz), listeners in their native language entrained to the phrase-level (2 Hz) and sentence level (1 Hz), even though these sub-harmonics were abstract linguistic structures and not part of the acoustic signal. Conversely, English listeners without Chinese knowledge did not track Chinese structures beyond the syllable. As such, these results show that (i) language knowledge influences tracking abilities and (ii) that cortical entrainment is involved in higher-level linguistic processing, rather than just responding to lower-level acoustic features such as detection of word onset or following a rhythm.

Thus, these data suggested that neural tracking of linguistic structures is quite automatic for native listeners and nonexistent for people unfamiliar with a language. However, this study only provided a categorical answer for the two extremes of the continuum (i.e., either full proficiency in a language or no knowledge of it), but not a quantitative answer of how much proficiency or comprehension of a language one needs for entrainment to occur. In a follow-up study, Blanco-Elorrieta et al. (2020) found that there is a continuum between proficiency in a second language and the extent to which listeners can track higher-order representations. Participants were Chinese-English listeners with different language proficiency levels in both languages, and the study used the same stimuli as Ding et al. (2016). These stimuli were embedded in four different noise conditions ranging from very noisy to fully clear speech. They found that bilingual listeners were able to track phrases and syllables in their native language (NL) at all levels of noise except during pure noise. However, in their second language (SL) they were always able to track syllabic rhythm but stopped tracking linguistic structures as soon as there was a significant level of noise (7.5 dB). Importantly, as proficiency increased, so did the tracking of the phrasal structures. In a similar vein, Lizarazu et al. (2021) showed that cortical tracking of natural speech in the delta-band is proportionate to the language proficiency (measured as years of instruction) of the listener, and Lu et al. (2023) showed that neural responses to higher-order linguistic structures (i.e., phrases and sentences) for L2 listeners was functionally related to an L2 subject’s language proficiency. Following these results, the authors in all three papers concluded that neural tracking enhances, and it might in fact be necessary to achieve, full comprehension of language. However, all studies quantified language proficiency by the years of instruction or via an offline language proficiency test, but didn’t directly measure language comprehension, which would be the type of direct evidence required to support the claim that there is a relation between the extent of the cortical tracking and language comprehension.

Here, we address that gap by examining neural tracking at different time scales using the English materials of Ding et al. (2016). Two groups were analysed, a group of native English listeners and a group of people who learned English as a second language. As an index of language comprehension in NL and SL, participants in both groups were tested in an independent sentence comprehension task (in English) with electroencephalography (EEG). This task had semantically congruent and semantically incongruent sentences, and in addition to their comprehension scores, we also measured the N400 as an online signature of language comprehension. We chose this index because previous research has shown that the peak of the N400 is sensitive to semantic violations that can only become apparent if language is understood (Kutas & Federmeier, 2011), and that it peaks differently for SL than NL subjects. Specifically, it either peaks later (Ardal et al., 1990; Hahne, 2001), or it has a smaller amplitude (Hahne & Friederici, 2001) in the SL than in the NL, positing it as a valid measure that indicates high-order language processing and comprehension.

In using this approach, this study seeks to inform the relationship between cortical entrainment and lexicosemantic processing. While both processes are considered key components of language comprehension, the interaction and interplay between them remains elusive (e.g., Molinaro et al., 2021, for a correlation between phase-locking and N400 effects associated to semantic predictability). If the extent of the tracking is indeed associated with language comprehension, it would follow to see a significant correlation in the SL group between peak amplitude and N400 magnitude (calculated out of the grand averaged event-related potential (ERP) results; semantically congruent/incongruent conditions). If, on the other hand, neural entrainment is a process fundamentally unrelated to language comprehension, we should expect no relation between both measures.

In sum, the present study used the N400 response to lexicosemantic incongruencies as an index of language comprehension and related it to the level of cortical entrainment to the frequencies corresponding to the linguistic hierarchies presented in the cortical tracking experiment: sentences, phrases, and syllables. Based on previous work (Ardal et al., 1990; Blanco-Elorrieta et al., 2020; Hahne, 2001; Hahne & Friederici, 2001), we predict that NL listeners will show a strong N400 component and will entrain to all linguistic structures. Conversely, SL listeners will probably show a reduced and perhaps delayed N400 component, and the empirical question is how that will relate to entrainment to abstract linguistic structures above the syllabic (purely rhythmic) level (i.e., to phrasal or sentential structures).

## MATERIALS AND METHOD

### Participants

Twenty-four native English listeners (11 women, mean age: 23.5, *SD*: 5.0, range: 18–38 years, 23 right-handed), and 26 Catalan/Spanish bilinguals that learned English as a second language at school (18 women, mean age: 21.9, *SD*: 2.6, range: 19–30 years, 24 right-handed) participated in the study. All participants were graduate or undergraduate students at Universitat Pompeu Fabra. They were recruited from the Center for Brain and Cognition (CBC) participant pool and received monetary compensation for their participation. Participants self-reported normal or corrected to normal vision, adequate hearing, no language or learning disabilities, no neurological/psychiatric disorders, and no current drug use. Before the experimental session, individual written consent was obtained from each participant.

We had five a priori criteria to exclude participants’ data: (i) technical issues during EEG acquisition such as saturation of the amplifiers, high electrode impedance, or electrode detachment; (ii) participant discomfort with the experimental set-up, expressed overtly by them or perceived by the experimenter; (iii) more than 25% of the EEG signal at any trial being irrecoverable due to large artifacts (e.g., laughing, sneezing, coughing, yawning, or body movements); (iv) keeping less than 75% of trials after pre-processing of the data due to noisy signal; and (v) scores below average level in the English test for the SL group. This resulted in six participants being excluded from our analyses. Thus, the final sample consisted of 22 English NL listeners (9 women, mean age: 23.5, *SD*: 5.0, range: 18–38 years, 21 right-handed), and 22 English SL listeners (14 women, mean age: 22.2, *SD*: 2.7, range: 19–30 years, 20 right-handed).

Native English speakers reported their proficiency to be at ceiling (10/10) in reading, writing, listening, and speaking. The SL speakers self-rated their proficiency as 8.1 out of 10 on average (*SD*: 1.0, range: 6.3–10). An independent samples *t* test was carried out in order to confirm that both groups were significantly different (M_NL_ = 10; M_SL_ = 8.1; *t*_(42)_ = 8.90, *p* = < 0.001). The English proficiency of the SL listeners was additionally evaluated via two standardized tests, assessing listening, grammar, and vocabulary abilities. To assess listening abilities, we administered a practice International English Language Testing System (IELTS) listening test (British Council, n.d.). Regarding grammar and vocabulary, evaluation was carried out using the *Straightforward Quick Placement & Diagnostic Test* (2012). Accuracy in the listening test was 86% (*SD* = 0.7), and 93% (*SD* = 0.05) in the grammar and vocabulary test. SLs additionally completed a Language History Questionnaire, an adaptation of Li et al. (2020) that also included questions about language exposure (Marian et al., 2007) and language usage in different settings (Luk & Bialystok, 2013). The mean age of acquisition for English was 4.9 (*SD* = 1.7).

### Stimuli

#### Entrainment experiment

For the entrainment task, materials consisted of 60 four-syllable sentences taken from Ding et al. (2016, supplementary table “English 4-syllable sentences”). Each sentence consisted of four monosyllabic words combined to form two-word noun phrases (adjective + noun) and two-word verb phrases (verb + noun). The combination of these two phrases resulted in four-word sentences (e.g., “big rocks block roads”). A 25 ms cosine window smoothed the offset of each syllable. Words were synthesized independently using the MacinTalk Synthesizer (male voice Alex, in Mac OS X 10.7.5). All syllables were adjusted to 320 ms in duration by padding or truncation, as in the original Ding et al. (2016) study (320 ms * 4 words per sentence = 1.28 s per sentence). Thus, the sentence rate was 1/1.28 Hz; the phrasal rate was 2/1.28 Hz, and the word rate was 4/1.28 Hz. Each trial consisted of the sequential presentation of 12 of these sentences. These trials were presented in random order to participants, and in total, each participant was exposed to 30 trials (*N* = 360 sentences).

#### N400 experiment

For the N400 task, 214 sentences recorded from 12 native English speakers (9 women) were taken from Foucart et al. (2015). Half of the sentences were semantically congruent, and the other half were semantically incongruent. The incongruent sentences were created by swapping the critical word from congruent sentences and were never placed at the end of the sentence (e.g., Congruent: “Sadly, I lost my *job* today”; Incongruent: “Sadly, I lost my *cave* today”). As detailed in Foucart et al. (2015), congruent and incongruent sentences differed in the cloze probability of the critical word, defined as the percentage of times it was used to complete the sentence (congruent, *M* = 7.6%, *SD* = 1.1; incongruent, *M* = 0%). Critical words in congruent and incongruent conditions were matched for age of acquisition (*p* = 0.43), frequency (*p* = 0.97), imageability (*p* = 0.62) and concreteness (*p* = 0.44).

Sentences were pseudorandomized and distributed into six lists, so the congruent–incongruent sentence pairs did not appear in the same list. Stimuli order were pseudorandomized, so participants did not hear the same speaker for more than three times in a row. Each participant was assigned to one of the six lists, containing 107 experimental sentences and seven practice sentences. Three out of the six lists contained 53 semantically congruent sentences and 54 semantically incongruent sentences. The reverse was true for the remaining lists.

### Procedure

Prior to the experimental session, participants of the SL group completed the IELTS Practice Listening Test to evaluate their auditory comprehension skills in their second language. After having completed the listening test, they were administered the MacMillan *Straightforward* Quick Placement & Diagnostic Test to evaluate their grammar and vocabulary proficiency. The completion of the tests took around 35 minutes. Both the NL and the SL groups completed the Language History Questionnaire (Li et al., 2020) to assess their linguistic background and language proficiency. Then the experimental session started, which was conducted in an electrically shielded and soundproof cabin to prevent noise interference. Cortical tracking and N400 experiments were conducted during the same experimental session, and the order of the tasks was counterbalanced across subjects.

In the tracking experiment, participants were presented with the synthesized speech sentences through headphones binaurally. After each trial (i.e., the presentation of 12 consecutive 4-syllable sentences), participants had to indicate how much they understood on a scale from 0 to 4 (0 being *nothing*, 4 being *everything*), via button press. In the N400 experiment, participants were auditorily presented with the experimental sentences. Following approximately 25% of the sentences, a yes/no comprehension question related to the content of the sentences was visually presented (for stimulus “At the salon I have a *perm* every weekend” the question was “Do you get the *perm* done every weekend?”) and participants had to answer yes/no via button press (∼1 question per 4 sentences). The questions were presented in pseudorandomised order, and they related to both congruent and incongruent sentences. The whole experimental session took approximately an hour and a half to complete.

### Data Acquisition and Analysis

#### Behavioral data

In the entrainment experiment, participants indicated with a button press how much they understood (4 = *everything*, 0 = *nothing*) from every set of sentences. The responses for each group (NL vs. SL) were submitted to a paired *t* test to determine whether the level of self-reported comprehension varied across groups. In the N400 experiment, it was the accuracy of the yes/no responses to the comprehension questions that was submitted to the same paired *t* test.

#### EEG data

EEG responses were continuously recorded with a 64-channel EEG system, digitized at a sampling rate of 500 Hz and referenced to the FCz channel. Three electrodes were used to record horizontal and vertical electrooculography (EOGs) and two reference electrodes were placed at the left and right mastoids. The impedance of the electrodes was kept below 30 kΩ. Forehead EEG channels that had a large correlation with the EOG components were discarded from the analysis and EOG artifacts were removed from the EEG using independent component analysis (ICA) decomposition. Additionally, EEG channels that tend to have loose contact or tend to be noisy (T7, T8, TP7, and TP8) were also removed from analysis. Entrainment data were analysed using FieldTrip (Oostenveld et al., 2011), an open-source Matlab toolbox, using custom-build Matlab code. ERP analysis of the sentence comprehension experiment was processed using BrainVision Analyzer (Brain Products Team, n.d.) software and custom-build Matlab routines.

#### Entrainment experiment

Since the frequencies of interest were in the low-frequency region, data were band-pass filtered from 0.1 to 25 Hz using a finite impulse-response (FIR) filter. Data were re-referenced offline to a common average reference (Ding et al., 2016). Subsequently, remaining bad channels were interpolated via triangulation using information from the neighboring channels. Next, trials were epoched from the beginning to the end of the auditory stimulus. To avoid the EEG transient response to sound onset, the first sentence of each trial was removed. Thus, trial duration was 14.08 s and trial frequency resolution was 0.071 Hz (1/14.08 Hz).

EEG data for each participant were first averaged across trials to obtain the time locked average activity and then in each trial were transformed into the frequency domain using the fast Fourier transform (FFT). The evoked power (i.e., the EEG responses synchronized to the speech information) was computed on the averaged activity across trials for each electrode and each participant. After performing the grand average over subjects, power was then averaged over electrodes. In order to account for the baseline differences in power at each frequency, we calculated the difference between power at each frequency with the averaged power at the four neighboring frequency bins.

The analysis of interest was performed in the sentential (1/1.28 Hz = 0.78 Hz), phrasal (2/1.28 Hz = 1.56 Hz) and syllable/word (4/1.28 Hz = 3.12 Hz) rate frequencies, by comparing the response amplitude at the peak frequency to the mean amplitude of the neighboring four frequency bins (two on each side) using a one-tailed paired *t* test for each group, as in Ding et al. (2016).

#### N400 experiment

Data were analyzed following commonly used pipelines in the N400 literature. Specifically, data were 0.1 high-pass filtered and 30 Hz low-pass filtered offline (Acunzo et al., 2012; Tanner et al., 2015) and re-referenced offline to the two mastoid channels. EEG responses were segmented into 800 ms epochs (−100 to 700 ms), time locked to the onset of the critical word of each sentence. Artifact rejection was applied for each trial when brain activity was above or below 75 μV (12.3% of trials were excluded on average). Baseline correction was applied with reference to pre-stimulus activity (−100 to 0 ms).

Trials were averaged across participants for each experimental condition (congruent/incongruent). To maximally capture the N400 effect, we focused our analysis on two time windows around 400 ms after the onset of the critical word, the 300–500 ms and 500–700 ms windows. The ERP mean amplitudes were computed from a selected region of interest (ROI), including 14 electrodes located at central and centroposterior sites (C5, C3, C1, Cz, C2, C4, C6, CP5, CP3, CP1, CPz, CP2, CP4, and CP6). For each time window and group of participants, a paired *t* test was conducted comparing semantically congruent and incongruent sentences. In addition, a paired-sample *t* test was performed at every sampling point and in each electrode to capture differences on the latency of the N400 effect in each group of participants. The false discovery rate was applied to correct for multiple comparisons.

#### Correlation analysis

Finally, for the SL group, a pairwise Spearman’s correlation analysis was conducted to explore the relation between (i) lexicosematic processing (indexed by the N400 effect; the difference between the semantically congruent and incongruent conditions), (ii) neural tracking (indexed by the amplitude of the EEG spectral peaks, sentential, phrasal, and syllabic rates), and (iii) language proficiency/comprehension measures (self-rated proficiency, the English listening test, and the behavioral data from the entrainment experiment). Data across variables were normalized through the min–max scaling method (caret library in R; Kuhn, 2008).

## RESULTS

### Behavioral Results

As expected, native speakers understood significantly more than non-native speakers. In the entrainment task, where participants indicated with a button press how much they understood (4 = *everything*, 0 = *nothing*), the NL group reported that they understood significantly more than the SL group (M_NL_ = 3.68; M_SL_ = 2.95; *t*_(42)_ = 3.35, *p* = 0.001). In the N400 experiment, the NL responded to questions more accurately than SL (M_NL_ = 95%; M_SL_ = 90%; *t*_(42)_ = 3.18, *p* = 0.002). These results showcase the better language comprehension abilities of the NL group than the SL group.

### Neural Entrainment Experiment

**Figure 1.**
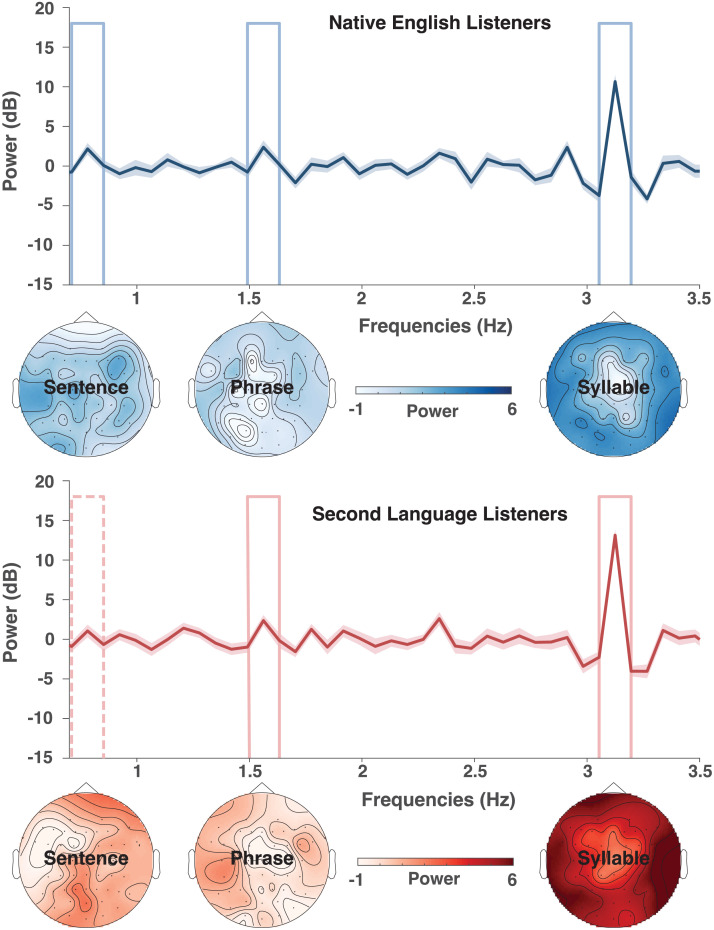
Electroencephalography (EEG) evoked power spectrum responses to sentences. Blue: native language (NL) group. Red: second language (SL) group. The bold lines show the grand average over subjects, the shaded area delimits the standard error of mean. Power for each person and electrode was converted into decibels. The three framed bins show the target frequencies at the sentential (0.78 Hz), phrasal (1.56 Hz), and syllabic (3.12 Hz) rates. The corresponding topography maps are plotted below. For the NL group, evoked power in all three target bins was significantly different from the neighboring bins. In contrast, the evoked power only produced significant peaks at the phrasal and syllabic rates for the SL group.

The native English listeners successfully tracked the syllabic, phrasal, and sentential rates, as per the power analysis at the sentential (*t*(21) = −1.84, *p* = 0.039), phrasal (*t*(21) = −2.72, *p* = 0.006), and syllabic frequency bins (*t*(21) = −5.16, *p* < 0.001; see Figure 1).

The SL group, however, only tracked the syllabic (*t*(21) = −5.85, *p* < 0.001) and phrasal (*t*(21) = −1.98, *p* = 0.030) structures, but the power of the peak corresponding to sentential rate (*t*(21) = −1.31, *p* = 0.101) was not significantly stronger than the power in the neighboring bins.

### N400 Experiment

**Figure 2.**
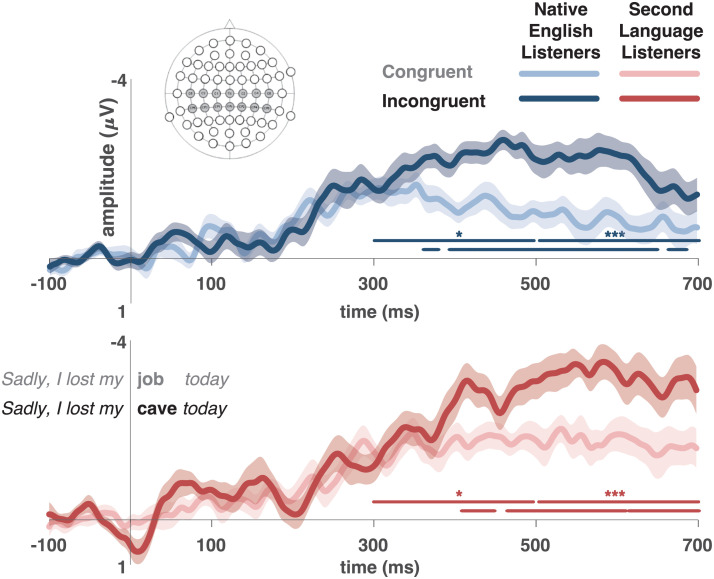
Grand averaged event-related potential (ERP) responses over central and centroposterior electrodes (C5, C3, C1, Cz, C2, C4, C6, CP5, CP3, CP1, CPz, CP2, CP4, and CP6). Blue: NL group. Red: SL group. The lighter line shows the responses for the semantically congruent condition, and the darker line shows the responses for the semantically incongruent condition. The shaded area delimits the standard error of mean. Time zero corresponds to the onset of the critical word of the sentence. Negativity is plotted up. Significant difference between semantic congruent and incongruent conditions in the two time windows of interest are marked with asterisks (*p < 0.05; **p < 0.01; ***p < 0.001). Significant differences for congruency at each time point (*p* < 0.05) are marked below the effect of the time windows.

The mean amplitude for the electrodes in the ROI was calculated in a [−100 to 700 ms] time window and averaged across participants and within conditions. Two statistical analyses were conducted. The first one considered the average ERP amplitude from semantically congruent and incongruent conditions in the selected ROI and two time windows of interest (300–500 ms and 500–700 ms). The second consisted of conducting a paired *t* test at every millisecond in the time window (see Figure 2).

### [300–500 ms] and [500–700 ms] Time-Window Analysis

For the NL group, semantically incongruent words elicited a larger negativity than congruent words in the two time windows of interest (300–500: *t*(21) = 2.8, *p* = 0.01; CI = 0.24–1.60; 500–700: *t*(21) = 4.3, *p* < 0.001; CI = 0.62–1.77). The SL group also detected the semantic incongruency, with semantically incongruent sentences eliciting a larger negativity than congruent ones (300–500: *t*(21) = 2.2, *p* = 0.04; CI = 0.03–1.34; 500–700: *t*(21) = 4.1, *p* < 0.001; CI = 0.73–2.22). As Figure 2 shows, the N400 effect was more prominent and started earlier for the NL group than for the SL group.

#### Relation between the N400 and neural tracking in SL

Previous research (e.g., Blanco-Elorrieta et al., 2020; Zoefel et al., 2018) has hinted at the possibility that neural tracking might allow for better comprehension of the speech input. This experiment set to test this idea by analyzing whether there is a relation between the N400 effect (taken as an electrophysiological signature of language comprehension) and the amplitude of the entrainment peaks at the syllabic, phrasal and sentential rates for SL. If the extent of the tracking is indeed associated with language comprehension, it would follow to see a significant correlation in the SL group between peak amplitude and both their proficiency level and N400 magnitude (calculated out of the grand averaged ERP results; semantically congruent/incongruent conditions). If, on the other hand, neural entrainment is a process fundamentally unrelated to language comprehension, we should expect no relation between both measures.

The results showed that the tracking of the sentential rate, correlated with the subjects’ language proficiency as measured by the Listening test (*r*(20) = 0.42, *p* = 0.051) as in Lizarazu et al. (2021) and Lu et al. (2023); most importantly, it was also correlated with both measures of online language comprehension—the N400 effect in the late time window (500–700; *r*(20) = −0.41, *p* = 0.058), and the comprehension rating during the neural entrainment experiment (*r*(20) = 0.46, *p* = 0.030)—suggesting that there is indeed a relation between cortical tracking and language comprehension. N400 effect did not correlate with any behavioral measure of comprehension.

## DISCUSSION

The current experiment set to evaluate the relation between (i) the ability to segment and identify linguistic structures and (ii) high-level semantic processing, as a means to clarify whether cortical tracking is a fundamental process for language comprehension. The majority of previous studies have used intelligibility manipulations to examine this topic, contrasting neural entrainment with speech and speech-like but unintelligible acoustic stimuli (i.e., speed changes to the speech: Ahissar et al., 2001; noise-vocoded speech: Peelle et al., 2013; Rimmele et al., 2015; time reversal: Gross et al., 2013; or the addition of background noise: Ding & Simon, 2013). The results obtained from this approach are mixed; with some research showing that there is a relation between entrainment and language comprehension, and others failing to find such effects (for a review, see Kösem & van Wassenhove, 2017).

Here, we took a different approach. We tested both a group of native English listeners and a group of Spanish/Catalan listeners who had learned English as a second language and thus varied in their language comprehension skills while keeping the auditory stimulus constant. The cortical tracking experiment showed that NL had better comprehension and better tracking of linguistic structures than the SL. Specifically, the NL group was able to track sentential, phrasal and syllabic structures; whereas SL listeners failed to track the most abstract syntactic structure, that is, the sentential rate. The semantic anomaly detection experiment showed that the NL group were more accurate at responding to the comprehension questions than the SL group (even though the SL group answered correctly in 90% of the cases). The N400 analysis showed that although both groups detected the semantic incongruencies, the effect started earlier and was more prominent for the NL group (as expected based on previous literature; see Kutas & Federmeier, 2011).

More interestingly for our purposes, there was a significant correlation between the N400 effect and the amplitude of the entrainment peak at the sentential rate of the entrainment experiment. The relationship between the variables indicates that as the N400 effect increases, indicating deeper semantic processing, so does the entrainment at the sentential level. Additionally, there was a significant correlation between entrainment at the sentential level and the behavioral measure of comprehension. Thus, our results suggest a meaningful relation between online language comprehension and cortical tracking of abstract linguistic structures.

This result goes contra recent evidence showing that an increase in the intelligibility of the speech does not result in increased tracking (Baltzell et al., 2017; Kösem et al., 2016; Kösem et al., 2023; Millman et al., 2015; Peña & Melloni, 2012; Zoefel & VanRullen, 2015b). In these studies, participants were presented with a degraded acoustic signal, but the experimenters manipulated comprehension by presenting them either with the degraded version or with the degraded version after having heard the clear version of the stimulus, which leads to increased comprehension. Importantly, however, in these studies (a) the comprehension of the speech was never full (even if comprehension increased after the presentation of the clear stimulus, it hovered around 60% maximum); and (b) the approach was categorical, there was no continuous degrading of the stimuli to capture incremental variation in comprehension or tracking.

By virtue of acquiring two continuous measures of comprehension, namely, the amplitude of the N400 and their behavioral comprehension scores, in addition to manipulating the profile of the listener as opposed to the stimulus, we uncovered a linear relationship between language comprehension and cortical tracking that can arguably only be explained within a framework that posits oscillations as not epiphenomenal to the acoustic signal but a fundamental process for language comprehension. This result is in line with the two other studies that have manipulated language comprehension as opposed to stimulus quality to address the relation between entrainment and language comprehension (Blanco-Elorrieta et al., 2020; Lizarazu et al., 2021). In both cases, an increase in language proficiency, and thus in language comprehension, came accompanied with significant increases in cortical tracking.

Thus, there seems to be a cohesive and increasing body of evidence coming from experiments manipulating comprehension abilities via a more naturalistic approach (e.g., listeners’ language proficiency) as opposed to artificial, unnatural manipulations of the auditory signal (e.g., spectral degrading, noise-vocoded speech), which shows that there is indeed a clear relation between language comprehension and cortical entrainment. In fact, our results suggest that cortical tracking becomes progressively attuned to the temporal structure of the second language (SL) as proficiency improves. This tuning may potentially underpin improved recognition of the distributional regularities in the SL, thus contributing to enhanced processing fluency in a newly acquired language.

## ACKNOWLEDGMENTS

We thank Albert Costa for the time, energy and joy that he brought to this project.

## FUNDING INFORMATION

Cristina Baus, Ministerio de Ciencia, Innovación y Universidades (https://dx.doi.org/10.13039/100014440), Award ID: RYC-2018-026174.

## AUTHOR CONTRIBUTIONS

**Cristina Baus**: Conceptualization: Supporting; Data curation: Supporting; Funding acquisition: Lead; Project administration: Lead; Writing – original draft: Equal; Writing – review & editing: Equal. **Iris Millan**: Conceptualization: Lead; Data curation: Lead; Formal analysis: Lead; Project administration: Supporting; Writing – original draft: Lead; Writing – review & editing: Supporting. **Xuanyi Jessica Chen**: Data curation: Supporting; Formal analysis: Lead; Project administration: Supporting; Visualization: Lead; Writing – review & editing: Supporting. **Esti Blanco-Elorrieta**: Conceptualization: Lead; Data curation: Supporting; Project administration: Supporting; Writing – original draft: Lead; Writing – review & editing: Equal.

## DATA AND CODE AVAILABILITY STATEMENT

Raw, and processed data, as well as data processing and analyses scripts are available from OSF public repository (https://osf.io/e6z3s/).

## TECHNICAL TERMS

Entrainment: Refers to the synchronization or alignment of neural oscillations with external stimuli. For example, if a rhythmic external stimulus, such as a visual or auditory stimulus, causes brain waves to synchronize with its frequency, it is said to induce entrainment.
Frequency Band: In the context of neural oscillations, a frequency band refers to a specific range of frequencies within the electromagnetic spectrum. Different frequency bands are associated with different cognitive functions and states of consciousness. Common frequency bands studied in neuroscience include delta (1–4 Hz), theta (4–8 Hz), alpha (8–13 Hz), beta (13–30 Hz), and gamma (30–100 Hz).
Cortical Tracking: Refers to the monitoring and recording of the neural activity in the cerebral cortex of the brain. This can be done using various neuroimaging techniques, such as electroencephalography (EEG) or magnetoencephalography (MEG), to observe and analyze the electrical or magnetic signals generated by neurons in specific regions of the cortex.
N400: An event-related potential (ERP) component that is often observed in electroencephalography (EEG) recordings. It is a negative-going waveform that peaks around 400 milliseconds after the presentation of a stimulus. The N400 is often associated with semantic processing in the brain and is sensitive to the meaningfulness or congruency of the presented stimulus within a given context.
Power: EEG measures the electrical activity generated by the neurons in the brain, and this activity can be categorized into different frequency bands. The power in each frequency band represents the strength or amplitude of the neural oscillations within that specific range.
